# Olfactory Responses to Natal Stream Water in Sockeye Salmon by BOLD fMRI

**DOI:** 10.1371/journal.pone.0016051

**Published:** 2011-01-17

**Authors:** Hiroshi Bandoh, Ikuhiro Kida, Hiroshi Ueda

**Affiliations:** 1 Division of Biosphere Science, Graduate School of Environmental Science, Hokkaido University, Sapporo, Japan; 2 Integrated Neuroscience Research Team, Tokyo Institute of Psychiatry, Tokyo, Japan; 3 Laboratory of Aquatic Ecosystem Conservation, Field Science Center for Northern Biosphere, Hokkaido University, Sapporo, Japan; Max-Planck-Institut für Neurobiologie, Germany

## Abstract

Many studies have shown that juvenile salmon imprint olfactory memory of natal stream odors during downstream migration, and adults recall this stream-specific odor information to discriminate their natal stream during upstream migration for spawning. The odor information processing of the natal stream in the salmon brain, however, has not been clarified. We applied blood oxygenation level-dependent (BOLD) functional magnetic resonance imaging to investigate the odor information processing of the natal stream in the olfactory bulb and telencephalon of lacustrine sockeye salmon (*Oncorhynchus nerka*). The strong responses to the natal stream water were mainly observed in the lateral area of dorsal telencephalon (Dl), which are homologous to the medial pallium (hippocampus) in terrestrial vertebrates. Although the concentration of L-serine (1 mM) in the control water was 20,000-times higher than that of total amino acid in the natal stream water (47.5 nM), the BOLD signals resulting from the natal stream water were stronger than those by L-serine in the Dl. We concluded that sockeye salmon could process the odor information of the natal stream by integrating information in the Dl area of the telencephalon.

## Introduction

Salmon have an amazing ability to migrate several thousands of kilometers from the ocean to their natal stream for spawning. For the long-distance open water migration, salmon might use visual cues and/or geomagnetic orientation [1], [2]. Hasler's group [3] has proposed the olfactory hypothesis to describe how salmon discriminate the natal stream from other rivers. Indeed, it has been accepted that juveniles imprint an olfactory memory of the natal stream odor during downstream migration, and adults recall the stream-specific odor for discriminating their natal stream during upstream migration. Many researchers have supported this hypothesis [4]–[7], and artificial chemicals (e.g., β-phenylethyl alcohol (PEA) or morpholine) as well as natural chemicals (e.g., dissolved free amino acid (DFAA)) have been applied for imprinting and discriminating experiments using coho salmon (*Oncorhynchus kisutch*) [8]–[11] and masu salmon (*O. masou*) [12]. Recent studies in our laboratory have suggested that DFAA in the natal stream water is a possible odorant substance for such chemical cues in anadromous chum salmon (*O. keta*) [13] and lacustrine sockeye salmon (*O. nerka*) [14]. Most of the studies mentioned above have utilized electrophysiological recordings, which can measure the temporal dynamics of odor information processing in small regions of the central nervous system. Because of the spatial limitations of electrophysiological techniques, however, the mechanisms underlying information processing of the natal stream odor in the central nervous system of salmon, especially in the telencephalon, has not been completely elucidated.

Functional magnetic resonance imaging (fMRI) is a non-invasive method that can measure the neuronal activity via changes in cerebral blood flow and metabolism [15]. Most fMRI studies have been used in cognitive and psychological fields in humans. However, fMRI has been developed to investigate brain functions in small animals, such as mice, songbirds and fish [16]–[21]. Some studies have used the blood oxygenation level-dependent (BOLD) fMRI technique to code odorant information and reveal spatial activity patterns of glomeruli in the main olfactory bulb of mice [16], [17]. In songbirds, BOLD fMRI was used to monitor auditory activation, which represented discrimination of sound properties in the telencephalon [18]. In carp (*Cyprinus carpio*), fMRI studies based on BOLD contrast and changes in cerebral blood volume have reported brain responses to fluctuations in ambient water temperature [19], [20]. These studies have verified that BOLD fMRI, at a high magnetic field, was able to map localized functional activities in small animals.

In the present study, BOLD fMRI was used to investigate natal stream odor information processing in the central nervous system by measuring the response to natal stream water in the olfactory bulb and telencephalon of lacustrine sockeye salmon. We established an *in vivo*, non-invasive fMRI method for salmon and recorded BOLD fMRI signals activated by odorant stimulation (either by natal stream water or L-serine). We analyzed areas in the telencephalon, described as the major target of secondary olfactory integration, in salmon that responded specifically to the natal stream water.

## Results

**Figure 1:**
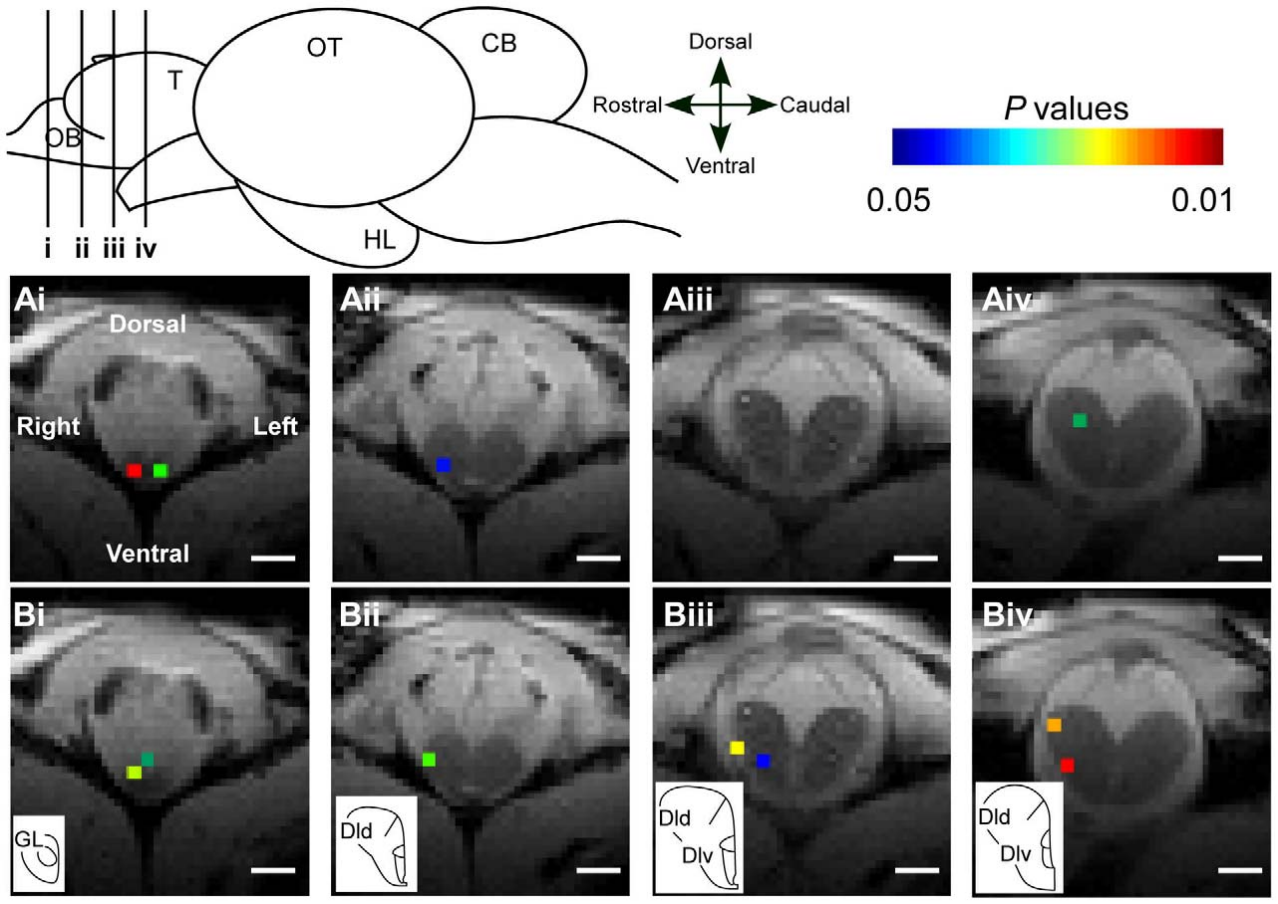
Blood oxygenation level-dependent (BOLD) functional magnetic resonance imaging (fMRI) maps of the same fish. A: L-serine; B: natal stream water. i: olfactory bulb; ii–iv: telencephalon. The activation map is defined by a threshold of P<0.05 corrected for multiple comparisons using false discovery rate approach. The BOLD signals are overlaid on the anatomical MRI image. Scale bar = 1.0 mm. CB, cerebellum; Dld, dorsal part of lateral area of dorsal telencephalon; Dlv, ventral part of lateral area of dorsal telencephalon; GL, glomerular layer; HL, hypothalamus; OB, olfactory bulb; OT, optic tectum; T, telencephalon.

Figure 1 shows the averaged functional images from six trials of the same fish defined by a threshold of *P*<0.05 corrected for multiple comparisons using false discovery rate approach. There were very few variations of activated areas among six fish. The activated pixels were elicited by a 3-min odorant stimulation of L-serine (Fig. 1*A*) or natal stream water (Fig. 1*B*) in the right olfactory epithelium. Except for one BOLD signal observed in the left hemisphere of the olfactory bulb, the BOLD signals were mostly detected in the right olfactory bulb and telencephalon (Fig. 1*A, B*). There were not many differences in activated areas between L-serine and the natal stream water in the olfactory bulb (Fig. 1*Ai* and 1*Bi*). In contrast, the activated extent in the telencephalon were clearly different between L-serine (Fig. 1*Aii–iv*) and the natal stream water (Fig. 1*Bii–iv*). Indeed, the BOLD signal response to the natal stream water was significantly higher than the response to L-serine (*P*<0.05). The distinct activations were mainly observed in dorsal (Dld) and ventral part (Dlv) of the lateral area of dorsal telencephalon (Fig. 1*Bii–iv*).

**Figure 2:**
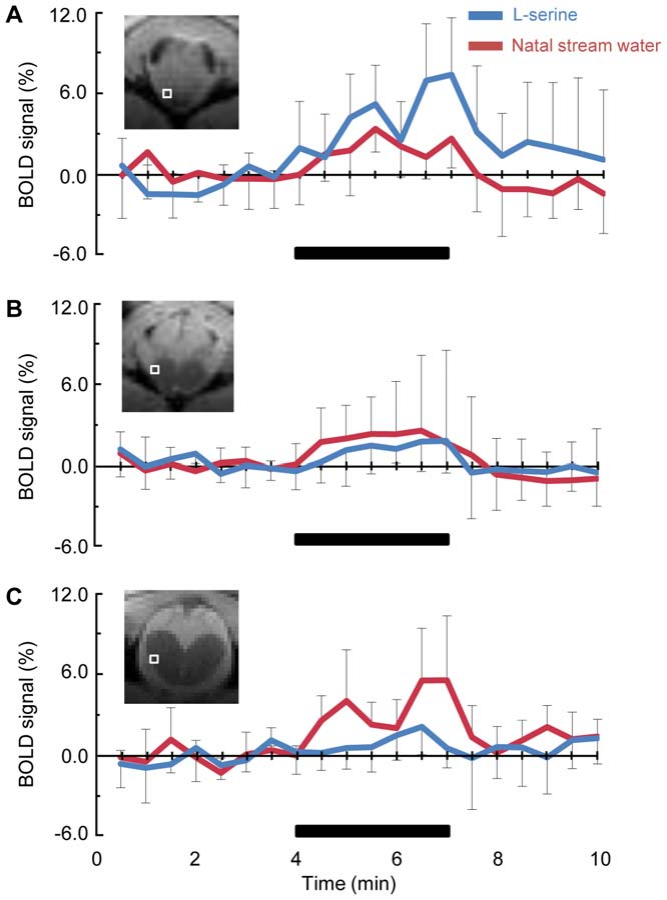
Time course of the BOLD signal changes induced by L-serine and natal stream water in the activation area. **A**: olfactory bulb corresponding to Fig. 1Ci; B: telencephalon corresponding to Fig. 1Cii; C: telencephalon corresponding to Fig. 1Civ. These responses are averaged for six stimulus paradigms from the same fish. The locations of each area are shown as boxes in the insert MRI images. The bars indicate the stimulus period (3 min).

Figure 2 reveals the time courses of the BOLD signals in the olfactory bulb and telencephalon of the sockeye salmon obtained by determining the activated area based on the Student's *t*-test value (*P*<0.05) that were averaged for six stimulus paradigms from the same fish. The increased BOLD signal correlated well with the on- and off-sets of both odorant stimulations. In the olfactory bulb, the BOLD signals by L-serine were stronger than those by the natal stream water (Fig. 2*A*). Although the concentration of L-serine was 20,000-times higher than that of total amino acid in the natal stream water, the BOLD signals by the natal stream water were stronger than those by L-serine in the Dlv part of the telencephalon (Fig. 2*B, C*). Some biphasic BOLD responses to L-serine and the natal stream water were observed in the olfactory bulb (Fig. 2*A*) and the Dlv part (Fig. 2*C*), respectively. During odorant stimulations for 3 minutes, some changes in the intensity BOLD response might occur. On the other hand, during resting period flowed by artificial fresh water (AFW) no changes in the BOLD signal were observed (Fig. 2).

## Discussion

Although there have been very few fMRI study in fish, in comparison with previous many electrophysiological studies with microelectrode, fMRI can measure simultaneously the wide range of neuronal activity in fish. This great advantage can provide various important data about information processing in the central nervous system of fish. We established an *in vivo* BOLD fMRI (7 Tesla) for salmon that survived for several hours under our fMRI set-up. We used BOLD fMRI technique to successfully map the olfactory bulb and telencephalon responses of salmon to natal stream water and L-serine. Natal stream water elicited stronger BOLD signals in the Dlv and Dld areas of the telencephalon. Although the L-serine water had a concentration of amino acid that was 20,000-times higher than natal stream water, L-serine did not elicit any strong responses in these areas. Some biphasic BOLD responses to L-serine and the natal stream water were observed in the olfactory bulb and Dlv area, respectively. Similar biphasic BOLD responses were reported in the mouse olfactory bulb [16]. The results of the present study suggested that odor information processing to natal stream water occurs in the telencephalon of sockeye salmon. A fluorescent carbocyanine dye (DiI) study indicated that the Dp and Dlv areas were the major target of secondary olfactory integration centers of salmonid dorsal telencephalon (pallial olfactory regions), and the Dld and Dm areas were also connected with the olfactory bulb [22]. An electrophysiological study on an odotopic map in the forebrain of channel catfish (*Ictalurus punctatus*) showed that lateral area of the telencephalon responded to amino acids and nucleotides [23]. Although the response of the olfactory bulb to the natal stream water had already been examined electrophysiologically in salmon [24]–[26], the distinct olfactory responses to the natal stream water in the Dlv and Dld areas of the telencephalon were clarified by the present BOLD fMRI.

Because there are big differences in the development of the telencephalon in teleosts (the eversion process) compared with higher vertebrates (the inversion process) [27], it is difficult to compare the structural homology of the telencephalon between teleosts and terrestrial vertebrates. Recent studies using a molecular marker in medaka (*Oryzias latipes*) showed that the telencephalic dorsal area and ventral area were homologous with the pallium and subpallium in mammals, respectively [28], [29]. These studies also proposed that the Dl area in teleosts were homologous to the medial pallium (hippocampus) in terrestrial vertebrates [23], [30], [31]. Studies involving a partial lesion of the Dl area have reported that it plays a crucial role in the acquisition of spatial learning [32], [33]. The Vv has been implicated in reproductive behavior in hime salmon, the same species as lacustrine sockeye salmon [34], and goldfish (*Carassius auratus*) [35]. However, no BOLD signal were detected in the Vv area in the present study after L-serine or natal stream water. The present findings on the olfactory response to the natal stream water in the Dl area of sockeye salmon were in accordance with neurohistochemical [22], molecular biological [28], [29] and behavioral studies [32], [33] suggesting that the Dl area have important functions in spatial memory and learning-related behaviors of teleosts.

Odor information is detected by a large number of odorant receptors in olfactory receptor neurons in the olfactory epithelium and sent to the olfactory bulb via the olfactory nerve. In the olfactory bulb, odor information is tuned by activation patterns of the glomeruli [16], [17], and it is conveyed to the higher central nervous system by the mitral cells, which are the secondary olfactory neurons in glomeruli. Calcium imaging reports built an odotopic map in the olfactory bulb of zebrafish (*Danio rerio*) by chemo-specificity projection of different odorant classes, such as amino acids, bile acids and nucleotides [36], [37]. Molecular biological studies, behavioral studies and two-photon Ca^2+^ imaging studies of odor responses in zebrafish have clearly shown that amino acids are detected by microvillus olfactory sensory neurons (OSNs), and these axons project to the glomeruli in the lateral olfactory bulb. Moreover, bile acids were detected in ciliated OSNs, and these axons projected to the glomeruli in the medial olfactory bulb [38], [39]. However, there has not been any information on the processing mechanisms of natal stream odor in the telencephalon of salmon.

Olfactory memory plays a key role in imprinting and recalling natal stream odor information in salmon. In mammals, studies on the formation of memory have recently been concentrated on the possible role of long-term potentiation (LTP) with a focus on *N*-methyl-_D_-aspartate (NMDA) receptors, which induce LTP [40]. It is believed that the hippocampus and amygdala play an important role in the formation of learning and memory in mammals. In teleost fish, LTP has also been described in the olfactory bulb of carp [41], [42] and the telencephalon of zebrafish [43]. Moreover, PEA-imprinted OSNs have significantly higher odor responses than non-PEA imprinted OSNs in the olfactory epithelium of coho salmon [10], [11], [44]. To date, studies have not determined if the olfactory memory of the natal stream is stored in the central or peripheral olfactory nervous system in salmon. Further neuroanatomical studies on partial lesioning of the Dl area as well as molecular biological studies on NMDA receptor gene expression levels in the Dl area, both of which are currently in progress in our laboratory, are necessary to clarify the olfactory memory processing in salmon.

## Materials and Methods

### Ethics statement

This study (20-2) was carried out following the “Guide for the Care and Use of Laboratory Animals in Field Science Center for Northern Biosphere, Hokkaido University” and Japanese Governmental Law (No. 105) and Notification No. 6, and was approved by the Committee of Laboratory Animals, Field Science Center for Northern Biosphere, Hokkaido University.

### Animal preparation

The present study used six 4-year-old lacustrine sockeye salmon (*Oncorhynchus nerka*) of both sexes, 23.6–27.4 cm in body length and 67.6–133.8 g in body weight, which were reared in the culture pond of the Toya Lake Station, Hokkaido University. There was no clear sexual difference in the BOLD fMRI experiments. The fish were transferred to Sapporo Salmon Museum, Hokkaido, Japan and were temporarily stored before the fMRI experiments.

**Figure 3:**
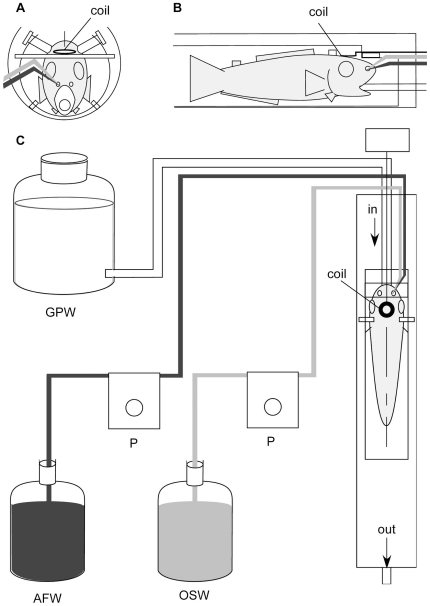
Schematic drawing of the animal-holding device containing an anesthetized sockeye salmon. A: frontal view; B: side view; C: water flow-through system mounted in the bore of the magnet during the magnetic resonance imaging experiment and the olfactory stimulus delivery system. The gill perfusion tube was fixed in the mouth of the fish. Odor stimulant water and AFW (artificial fresh water) water perfusion tubes were fixed in the right nasal cavity. coil, ^1^H transmit/receive surface-coil (diameter: 5 mm); OSW, odor stimulant water; GPW, gill perfusion water; P, peristaltic pump.

Each sockeye salmon was anesthetized by putting FA100 (eugenol; Tanabe Seiyaku Co. Ltd, Osaka, Japan) at a concentration of 0.5 ml/l in tap water they were swimming in, and immobilized by intramuscular injection of gallamine triethiodide (Sigma Chemical Co., St Louis, USA) at a concentration of 3 mg/kg body weight. The fish were stabilized on a holding device, using a pair of orbital ridge acrylic clamps, wrapped in a wet Kim-Wipe and maintained in a flow-through system with perfusion tubes into their mouth by gill perfusion at 400 ml/min (Fig. 3). The water temperature of the gill perfusion water, which was tap water, was kept at 13°C by regularly adding ice.

**Figure 4:**
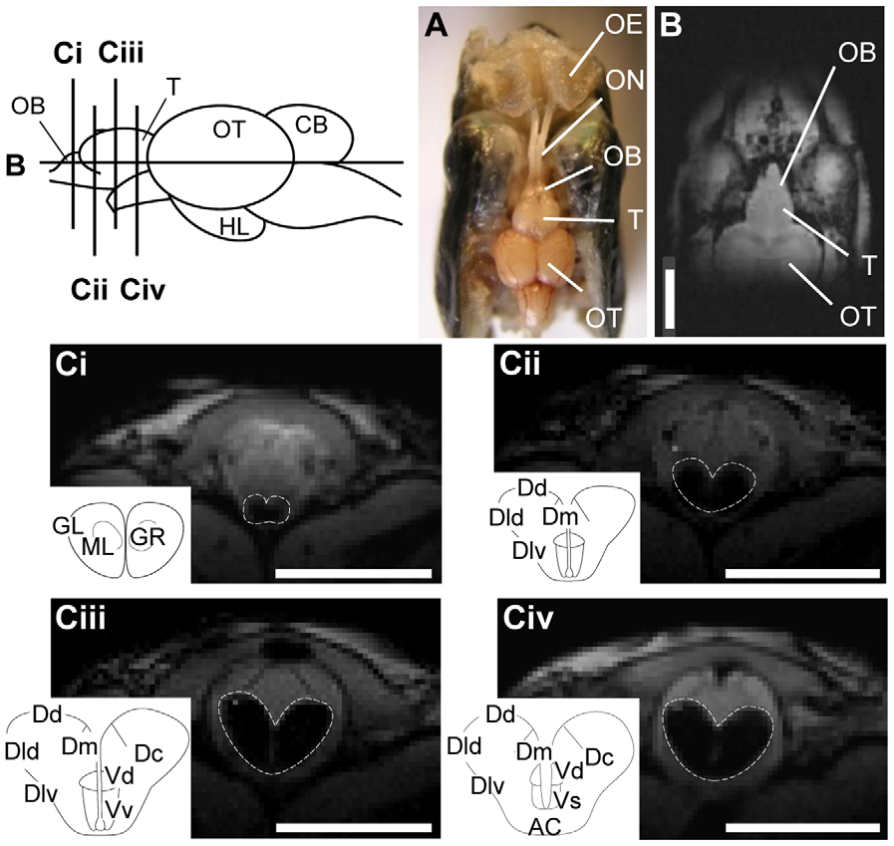
Magnetic resonance imaging (MRI) scan and dissected view of the front part of a lacustrine sockeye salmon head. *A*: Dissected view; *B*: MRI horizontal section; *Ci–iv*: serial MRI scan of frontal sections of the olfactory bulb (*Ci*) and the telencephalon (*Cii–iv*). The olfactory bulb and telencephalon are outlined by dotted lines. Scale bars = 5.0 mm. AC, anterior commissure; CB, cerebellum; Dc, central area of dorsal telencephalon; Dd, dorsal area of dorsal telencephalon; Dld, dorsal part of lateral area of dorsal telencephalon; Dlv, ventral part of lateral area of dorsal telencephalon; Dm, medial area of dorsal telencephalon; GL, glomerular layer; GR, granular layer; HL, hypothalamus; ML, mitral cell layer; OB, olfactory bulb; OE, olfactory epithelium; ON, olfactory nerve; OT, optic tectum; T, telencephalon; Vd, dorsal area of ventral telencephalon; Vs supracommissural area of ventral telencephalon; Vv, ventral area of ventral telencephalon.

The heads of sockeye salmon were dissected from the olfactory epithelium to the cerebrum (Fig. 4*A*), and the olfactory bulb and telencephalon were non-invasively observed by horizontal imaging of the front part of the head (Fig. 4*B*). The serial frontal sections of the olfactory bulb (Fig. 4*Ci*) and telencephalon (Fig. 4*Cii–iv*) were clearly visualized by T_1_-weighted anatomical MRI images. The middle of olfactory bulb and the anterior part of the telencephalon were comparable across experimental fish, but the anterior part of the olfactory bulb and the posterior part of the telencephalon (Dp) were excluded from the analysis because the image positions were slightly different between individual fish. In a cytoarchitectural study of forebrain organization in rainbow trout (*O. mykiss*) [22], the telencephalon was divided into dorsal (D) and ventral areas (V). The D area was subdivided into the central (Dc), dorsal (Dd), lateral (Dl) and medial areas (Dm), and the Dl area was further divided into the dorsal (Dld) and ventral parts (Dlv). The V area was further subdivided into the dorsal (Vd) and ventral areas (Vv) (the insert schematic drawing in Fig. 4*Cii–iv*).

### Odorant stimulation

The spring water of the Lake Toya Station, Hokkaido University was used as the natal stream water because the lacustrine sockeye salmon were reared in this spring water since hatching. The chemical compositions of natal stream water were reported to contain various amino acids, and the total concentration of amino acids was 47.5 nM (Table S1 for the first supporting information table) [12]. Water containing L-serine (1 mM), which has been shown to be a potent odor substance for teleosts [45], [46], was used as the control stimulus water. To protect the epithelium from desiccation and thoroughly rinse the olfactory organ, artificial fresh water (AFW: 0.5 mM NaCl, 0.05 mM KCl, 0.4 mM CaCl_2_, and 0.2 mM NaHCO_3_, pH 7.3) was constantly flowing over the olfactory epithelium during all times except the period of odorant stimulation. The odorant stimulus solutions and AFW were delivered though polyethylene tubes into the right olfactory rosette at the rate of 10 ml/min for 3 min with a peristaltic pump (Micro Tube Pump MP-3, Tokyo Rikakikai Co., Ltd., Tokyo, Japan) (Fig. 4). L-serine and the chemicals for the AFW were purchased from Wako Pure Chemical Industries, Tokyo, Japan.

The stimulation paradigms for one-block design were employed. An fMRI experiment required 10 min consisting of a pre-stimulation (AFW, 4 min), an odorant stimulation (the natal stream water or L-serine, 3 min) and a post-stimulation rest period (AFW, 3 min). The interval between successive stimulations was ∼10 min. The stimulation paradigm was repeated six times for each odorant.

### fMRI experiments

All the fMRI data were obtained using a horizontal 7 Tesla magnet interfaced to a Varian Unity INOVA console (Varian Inc., CA, USA) with a circular radio-frequency transmit-receive surface coil. The fMRI data were acquired using a multi-slice gradient-echo imaging sequence with the following scan parameters: repetition time/echo time = 470/10 ms, slice thickness = 1 mm, field of view = 20 mm×20 mm, matrix size = 64×64 for five slices. The T_1_-weighted anatomical images were obtained with variable inversion recovery weighting per slice. We used the following imaging parameters: image dimension = 128×128 pixels; field of view = 20 mm×20 mm; slice thickness = 1.0 mm; repetition delay = 5.0 s; echo time = 10 ms). The inversion recovery time was selected to be maximum contrast between the telencephalon (or the olfactory bulb) and the surrounding tissues.

### Data analysis

The BOLD fMRI data were processed using MATLAB software (MathWorks, Inc., MA) and an in-house written software. For each BOLD fMRI experiment, the Student's *t*-test was performed on a pixel-by-pixel basis using 14 images obtained during rest and 6 images obtained during stimulus conditions. Statistically significant pixels were considered at a level of *P*<0.05 corrected for multiple comparisons with false discovery rate approach [47]. The activation pixels were overlaid onto the corresponding anatomical MRI image. The time courses of the BOLD signals in the olfactory bulb and telencephalon of the sockeye salmon were obtained by determining the activated area based on a level of *P*<0.05 corrected for multiple comparisons that were averaged for six stimulus paradigms from the same fish. Images with head-movement artifacts, which were identified by a center-of-mass analysis [16], [17], were not considered for further analyses. The time course of the BOLD signals was represented as mean ± standard deviation (SD).

## Supporting Information

Table S1 The concentration of amino acids in the spring water of Toya Lake Station analyzed by Shoji et al. (2000) [12].
(PDF)
