# Supplementary material for: Olfactory Responses to Natal Stream Water in Sockeye Salmon by BOLD fMRI
Source: PLoS One. 2011 Jan 17;6(1):e16051. doi: 10.1371/journal.pone.0016051 (PMC3022028; doi:10.1371/journal.pone.0016051)
Supplement: Table S1 — The concentration of amino acids in the spring water of Toya Lake Station analyzed by Shoji et al. (2000) [12]. (PDF) [file pone.0016051.s001.pdf]

Table S1. The concentration of amino acids in the spring water of Toya Lake Station

Amino acids	Concentration (nM)
o-Phosphoserine	8.9
Taurine	5.6
o-Phosphoethanolamine	1.7
L-Aspartic acid	0.3
L-Threonine	5.2
L-Serine	2.3
L-Glutamic acid	2.0
L-Arginine	0.8
Glycine	5.0
L-Alanine	3.0
L-Valine	1.1
L-Isoleucine	0.8
L-Tyrosine	0.6
L-Phenylalanine	0.6
β-Alanine	1.5
γ-Amino butyric acid	0.4
L-Ornithine	2.2
L-Lysine	1.2
L-Histidine	0.9
L-Anserine	1.9
L-Cysteine	0.4
L-Glutamine	1.1
**Total**	**47.5**
